# Impact of a 2-Day Critical Care Ultrasound Course during Fellowship Training: A Pilot Study

**DOI:** 10.1155/2015/675041

**Published:** 2015-08-05

**Authors:** Vi Am Dinh, Paresh C. Giri, Inimai Rathinavel, Emilie Nguyen, David Hecht, Ihab Dorotta, H. Bryant Nguyen, Ara A. Chrissian

**Affiliations:** ^1^Division of Pulmonary and Critical Care, Department of Medicine, Loma Linda University, Loma Linda, CA, USA; ^2^Department of Emergency Medicine, Loma Linda University, Loma Linda, CA, USA; ^3^School of Medicine, Loma Linda University, Loma Linda, CA, USA; ^4^Division of Critical Care, Department of Anesthesia, Loma Linda University, Loma Linda, CA, USA

## Abstract

*Objectives*. Despite the increasing utilization of point-of-care critical care ultrasonography (CCUS), standards establishing competency for its use are lacking. The purpose of this study was to evaluate the effectiveness of a 2-day CCUS course implementation on ultrasound-naïve critical care medicine (CCM) fellows. *Methods*. Prospective evaluation of the impact of a two-day CCUS course on eight CCM fellows' attitudes, proficiency, and use of CCUS. Ultrasound competency on multiple organ systems was assessed including abdominal, pulmonary, vascular, and cardiac systems. Subjects served as self-controls and were assessed just prior to, within 1 week after, and 3 months after the course. *Results*. There was a significant improvement in CCM fellows' written test scores, image acquisition ability, and pathologic image interpretation 1 week after the course and it was retained 3 months after the course. Fellows also had self-reported increased confidence and usage of CCUS applications after the course. *Conclusions*. Implementation of a 2-day critical care ultrasound course covering general CCUS and basic critical care echocardiography using a combination of didactics, live models, and ultrasound simulators is effective in improving critical care fellows' proficiency and confidence with ultrasound use in both the short- and long-term settings.

## 1. Introduction

Point-of-care ultrasonography is a rapidly developing field embraced by multiple medical specialties including primary care, emergency medicine, and critical care medicine departments [1]. Common point-of-care critical care ultrasound (CCUS) applications include assessing the vascular, cardiopulmonary, and abdominal systems, as well as guiding invasive procedures and hemodynamic management [1–3]. With the increased availability and practicality of bedside ultrasound, intensivists worldwide are incorporating this technology into regular practice [4–6].

Despite the rapid increase of point-of-care ultrasound use in the ICU and the recognition by critical care fellowship programs for the need of formal CCUS training programs, standardized education does not yet exist [7, 8]. Even though CCUS competency requirements for image acquisition and interpretation have been outlined [9–11], there remains no consensus on how the education, training, and evaluation of these competencies should be achieved [12]. Consequently, CCUS education during fellowship can be inconsistent between training programs and may lead to varied ultrasound proficiency among graduating fellows and practicing intensivists [1, 11, 13]. This creates the risk of inappropriate ultrasound utilization in critical situations.

The implementation of an ICU ultrasound curriculum based on published guidelines and positive evidence-based educational outcomes is important for the standardization of training among critical care fellowships [9, 10]. Herein, we present such a course and hypothesize that it will serve as a valid educational tool for critical care ultrasonography. The purpose of this study is to evaluate the short- and long-term impact of a formal 2-day critical care ultrasound course during fellowship training on critical care fellows' ultrasound knowledge, skills, and attitudes.

## 2. Materials and Methods

### 2.1. Study Design and Setting

This was a prospective observational cohort study examining the effect of a 2-day CCUS course on ICU fellows from August 1, 2013, to November 30, 2013, at a university-based tertiary care medical center. Inclusion criteria were that the subject needed to be currently enrolled in a medical ICU (MICU) or surgical ICU (SICU) fellowship at the study institution. Subjects were excluded if they had any ultrasound certification or attended a formal CCUS course within the previous 12 months. A total of eight ICU fellows (6 MICU and 2 SICU) were enrolled in the study. Subjects were used as self-controls and examined at three predetermined time points defined as PRE-CCUS (within 1 week prior to CCUS course), POST-CCUS (within 1 week after CCUS course), and 3MO-CCUS (3 months after CCUS course). The study was approved by the Institutional Review Board and was considered to present minimal risk to the subjects. Subjects were not notified of any of the test answers or scores until after study completion.

We aimed to assess the effect of our course content and delivery on learners using the validated Kirkpatrick 4-level model of evaluating training outcomes [14, 15]. The first level, effects on “reaction,” was examined in the form of surveys assessing comfort level of scanning. Level 2, effects on “learning,” was assessed by scores on written, image acquisition, and pathologic image interpretation tests. Level 3, effect on “behavior,” was assessed by comparing the number of learner scans performed 3 months after the course to the number of reported scans immediately before the course. Finally, Level 4, “results,” was assessed by evaluating retention scores on written, image acquisition, and pathologic image interpretation tests 3 months after the course.

### 2.2. Course Curriculum

The 2-day course (Table 1) included both general CCUS and basic critical care echocardiography (CCE) with content consistent with national guidelines [9, 10]. The format of the course was multimodal and consisted of 1.5–2 hours of lectures, a live demonstration on the lecture topic by an expert lecturer, and a focused session on the lecture topic with ultrasound practicum on live healthy model volunteers, followed by learning pathologic image interpretation with cases using an ultrasound simulator (SonoSim Ultrasound Trainings Solution, Santa Monica, CA). The live demonstration by the lecturer consisted of using 2 projectors, with one projector connected to the ultrasound machine showing a real-time ultrasound image and with the other projector simultaneously connected to a camcorder projecting the instructor's hand position, movements, and transducer manipulation on a live model.

### 2.3. Course Assessment

#### 2.3.1. Ultrasound Knowledge: Written Test

To assess CCUS knowledge and retention, each ICU fellow completed a 50-question test during the PRE-CCUS, POST-CCUS, and 3MO-CCUS time points. The question distribution was five physics, fifteen cardiology, twelve pulmonary, fourteen abdominal, and four vascular domains. To minimize recall bias, the test questions were randomly reordered at each of the study time points. CCUS instructors, with formal ultrasound certification as registered diagnostic medical sonographer (RDMS), registered diagnostic cardiac sonographer (RDCS), and/or Advanced Perioperative Transesophageal Echocardiography (PTEeXAM), developed the physics, cardiology, abdominal, and vascular questions. The director of interventional pulmonology with significant lung ultrasound experience developed the pulmonary questions. All questions were peer-reviewed amongst the CCUS instructors prior to final distribution.

#### 2.3.2. Normal Image Acquisition on Healthy Volunteers

To assess ultrasound image acquisition ability, all ICU fellows were evaluated by a CCUS faculty instructor during live scanning of a healthy model patient during the PRE-CCUS, POST-CCUS, and 3MO-CCUS time points. Fellows were randomly assigned to the faculty instructor; however, the same faculty tested the fellows at all time points. The test consisted of an 84-point checklist, and organ systems tested were abdominal, pulmonary, vascular, and cardiac. The test evaluated the subject on proper patient positioning, correct use of the machine and transducers, ability to acquire standard ultrasound images, and quality of image acquisition.

#### 2.3.3. Pathologic Image Interpretation with Ultrasound Simulator

To assess pathologic image interpretation, all ICU fellows were tested on the ultrasound simulator using 4 cases during the PRE-CCUS, POST-CCUS, and 3MO-CCUS time points. Each case had 20 questions testing the following organ systems: abdominal, pulmonary, vascular, and cardiac. There was a possible total of 80 points for all four cases. Case 1 was a patient with decompensated congestive heart failure with severely depressed ejection fraction and pulmonary edema. Case 2 was a patient with a large pericardial effusion and tamponade. Case 3 was a trauma patient with hemoperitoneum, right hemothorax, and left pneumothorax. Case 4 was a patient with massive pulmonary embolism with findings of right ventricular dilation/hypokinesis, noncollapsible IVC, and right lower extremity deep vein thrombosis.

#### 2.3.4. Survey on Ultrasound Comfort Level and Use

To assess the changes in reaction and behavior, all ICU fellows completed a 16-question survey during the PRE-CCUS, POST-CCUS, and 3MO-CCUS time points. The questions assessed how many self-reported scans the fellows performed each week in the ICU as well as comfort level of specific CCUS applications including general CCUS, abdominal ultrasound, pulmonary ultrasound, lower extremity vascular ultrasound, echocardiography, ultrasound-guided central line, ultrasound-guided peripheral line, and ultrasound-guided thoracentesis.

### 2.4. Data Collection

All fellows were given a subject code number for deidentification for data analysis. Data collected from surveys and pre/posttests were transferred to electronic format using an online secure resource (Qualtrics.com). The site is password protected and data is only available to authorized study personnel.

### 2.5. Statistical Analysis

Student's *t*-test, chi-square, or ANOVA with repeated measures was performed to determine the changes in performance skills, test scores, and survey results between precourse, postcourse, and 3-month follow-up. Data analysis was performed using STATA 13.1.

## 3. Results

Six MICU and two SICU fellows were enrolled in the study. All eight fellows completed 100% of the surveys and tests at all three assessment time points. Results are expressed as percentage ± standard deviation. *p* values convey significance when compared to the PRE-CCUS group. There were no statistically significant differences among baseline results between the 1st-, 2nd-, and 3rd-year fellows (Table 2).

### 3.1. Ultrasound Knowledge: Written Test

**Figure 1:**
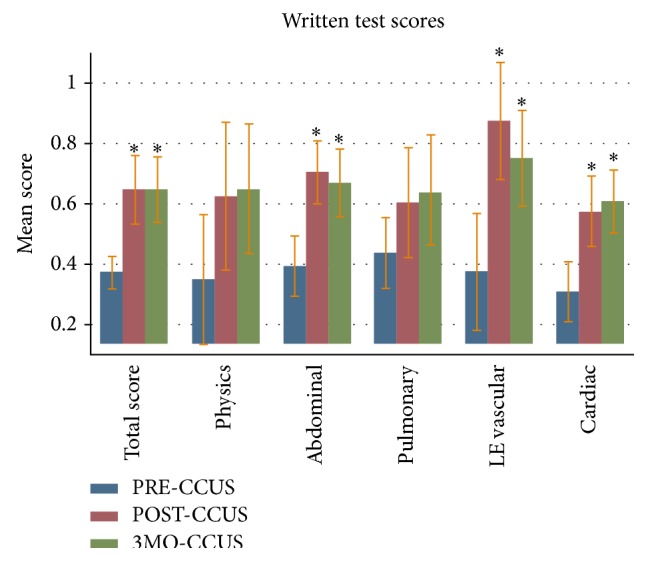
Written test score results with mean scores and standard deviation. “PRE-CCUS” group was tested within 1 week before CCUS course, “POST-CCUS” group was tested within 1 week after CCUS course, and “3MO-CCUS” group was tested 3 months after CCUS course. LE: lower extremity.  ^*∗*^Statistical significance when compared to the PRE-CCUS group.

The total written test score in the PRE-CCUS group was 37.3%  ± 6.3%. The total written test scores in the POST-CCUS and 3MO-CCUS groups were 64.8%  ± 13.6% (*p* < 0.05) and 64.8%  ± 12.9% (*p* < 0.05), respectively. Analysis of the five components of the test showed a statistically significant increase in scores of the POST-CCUS and 3MO-CCUS groups with respect to the following three organ systems: abdominal, lower extremity vascular, and echocardiography (Figure 1). Nonsignificant increase in score was noted in the physics and pulmonary components.

### 3.2. Normal Image Acquisition on Healthy Volunteers

**Figure 2:**
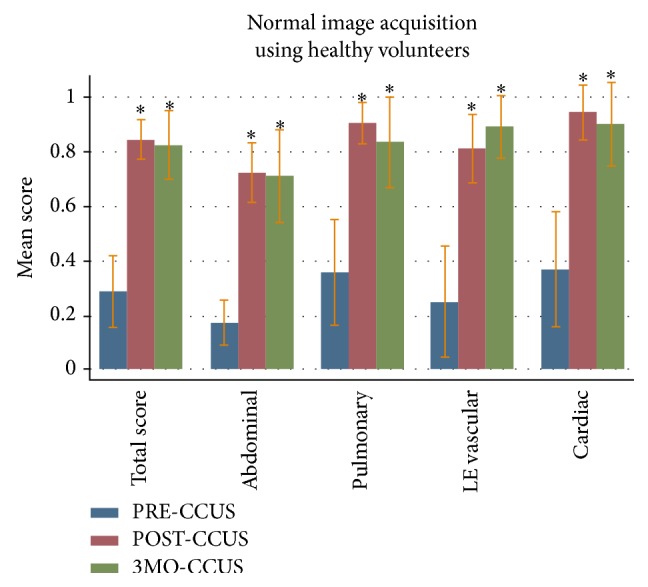
Normal image acquisition test score results with mean scores and standard deviation. “PRE-CCUS” group was tested within 1 week before CCUS course, “POST-CCUS” group was tested within 1 week after CCUS course, and “3MO-CCUS” group was tested 3 months after CCUS course. LE: lower extremity.  ^*∗*^Statistical significance when compared to the PRE-CCUS group.

The total image acquisition score in the PRE-CCUS group was 28.6%  ± 15.5%. The total image acquisition scores in the POST-CCUS and 3MO-CCUS groups were 84.1%  ± 8.5% (*p* < 0.05) and 81.9%  ± 15.0% (*p* < 0.05), respectively. In addition, when compared to the PRE-CCUS group, scores in all individual organ systems tested (abdominal, pulmonary, lower extremity vascular, and echocardiography) were significantly higher in the POST-CCUS and 3MO-CCUS groups (Figure 2).

### 3.3. Pathologic Image Interpretation

**Figure 3:**
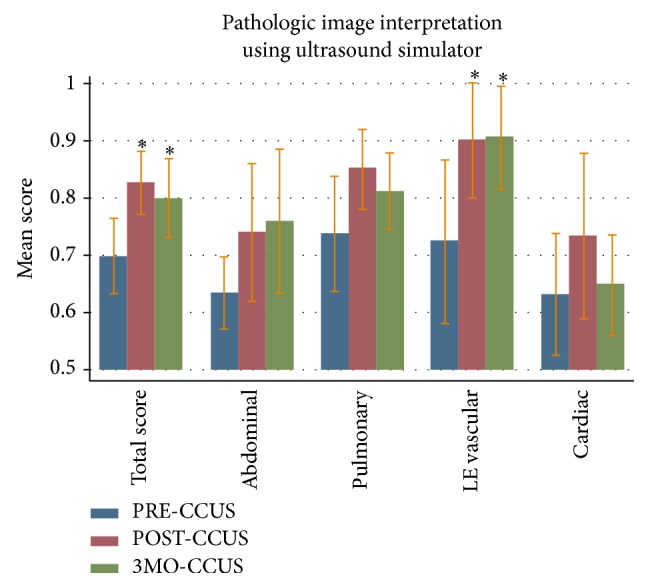
Pathologic image interpretation test score results with mean scores and standard deviation. “PRE-CCUS” group was tested within 1 week before CCUS course, “POST-CCUS” group was tested within 1 week after CCUS course, and “3MO-CCUS” group was tested 3 months after CCUS course. LE: lower extremity.  ^*∗*^Statistical significance when compared to the PRE-CCUS group.

The total pathologic image interpretation score in the PRE-CCUS group was 69.9%  ± 7.9%. The total pathologic image interpretation scores in the POST-CCUS and 3MO-CCUS groups were 82.7%  ± 6.6% (*p* < 0.05) and 80.1%  ± 8.2% (*p* < 0.05), respectively. Compared to the PRE-CCUS group, there was also a statistically significant increase in scores of the POST-CCUS and 3MO-CCUS groups in the lower extremity vascular scores (Figure 3).

### 3.4. Attitudes and Behavior: Questionnaire

Comfort level and self-reported scans regarding CCUS were surveyed using a 16-point questionnaire at the three time points. Comfort level increased significantly in the POST-CCUS and 3MO-CCUS groups in general bedside ultrasound, abdominal ultrasound, pulmonary ultrasound, lower extremity vascular ultrasound, and echocardiography (Table 3). There was a significant increase in the number of self-reported scans at 3 months in general bedside ultrasound, pulmonary ultrasound, lower extremity vascular ultrasound, and echocardiography (Table 4).

## 4. Discussion

Our study was a self-evaluation of a critical care ultrasound course taught by a multidisciplinary team of expert instructors. We implemented a formal 2-day CCUS course for critical care fellows that met published societal requirements for basic CCUS competency [9, 10]. Our results showed that such a course could improve skills in ultrasound knowledge, normal image acquisition, pathologic image interpretation, and comfort with ultrasound technique. These improvements were apparent immediately after the course, persisted 3 months after, and led to increased ultrasound usage in clinical practice. Consequently, we believe our course represents one possible model of implementing CCUS education and provide evidence that it might be a useful launch pad for medical staff wishing to start using ultrasound in their daily practice.

Several obstacles may be contributing to lagging CCUS education in the United States [6–8]. These include the lack of regulation in training requirements, inconsistent formatting of course delivery, variable focus and breadth of educational material, and a paucity of proficient faculty to provide instruction. Furthermore, the potential educational benefits of many proposed training programs have not been validated [1, 13, 16–18]. To help establish more uniform CCUS training and enhance the ultrasound proficiency of practicing intensivists, implementation of a course rooted in evidence-based, learner-oriented outcomes may be helpful.

We used a comprehensive method of self-assessment, Kirkpatrick's four-level training evaluation model, to highlight the positive educational impact of our course [14, 15]. To judge the first level, the “reaction” of our students to the course, we demonstrated their enhanced comfort with ultrasound technique after taking our course. The second Kirkpatrick level, “learning,” was judged by showing immediate postcourse improvements on several levels, including theoretical ultrasound knowledge, normal image acquisition, and pathologic image interpretation (Figures 1–3). By reporting increased ultrasound usage during their ICU rotations, our students confirmed the positive impact of our course on the third Kirkpatrick level, “behavior” (Table 4). To evaluate the fourth and perhaps most important Kirkpatrick level, “results,” we assessed the persistence of level 2 learned material. Indeed, our trainees showed both acquired theoretical ultrasound knowledge and practical skill maintained three months after course (Figures 1–3). This was a novel finding, since most other studies have only reported on immediate benefits of their training programs [19]. In short, our critical care ultrasound course seemed to have consistent and lasting beneficial effects across all areas deemed important for an effective training program, according to the Kirkpatrick system.

Although this was a pilot study involving a relatively small group of subjects, it was comprehensive in scope. The content and time spent on each topic adhered to published guidelines [9, 10]. More importantly, in contrast to previous investigations that highlight educational effects of programs focusing on specific organ systems such as critical care echocardiography on ultrasound-naïve trainees [19–22], we utilized a more inclusive model. The rapidly expanding critical care ultrasound literature overwhelmingly supports a multisystem approach to the evaluation and management of the critically ill patient. Established protocols suggest the integration of bedside pulmonary, cardiac, abdominal, and vascular ultrasound to quickly assess patients in acute respiratory and/or circulatory failure and to combine the results in formulating management plans [23–27]. We organized our course around these major organ systems, while also teaching, in parallel, the ultrasound applications for corresponding common procedures in the ICU (Table 1). Limited studies exist on looking at comprehensive CCUS training, with one study examining the benefits of a web-based curriculum covering general CCUS without CCE using a web-based and simulation format, while another study looked at the educational value of a 6-week general CCUS and basic CCE course [28, 29]. Our study differs because we believe that a CCUS course should include echocardiography given its benefit in the evaluation of unstable ICU patients. In addition, it may not be feasible to have a 6-week ultrasound curriculum at many institutions given the varied schedules of critical care fellows. The use of a multidisciplinary instruction faculty, with a varied ultrasound background and expertise, also helped create a well-rounded experience for the course attendees. Furthermore, this “wide-net” approach may aid other institutions in establishing similar courses. Since recent data suggest only 7–33% of faculty teaching in academic CCM fellowships are trained in CCUS [7], recruiting potential ultrasound instructors may need to target a range of disciplines, including radiology, emergency medicine, cardiology, pulmonology, surgery, and cardiothoracic anesthesia.

Another novel aspect of our course was the use of an ultrasound simulator to teach pathologic image interpretation and acquisition. It is difficult to incorporate and standardize live pathology into a course given the impracticality of finding and recruiting patients with specific disease states. Some ultrasound courses use case-based group presentations in which faculty present clinical scenarios and corresponding ultrasound findings to groups of learners [30]. While this approach may be resource-efficient, memory retention has been shown to be low and students are deprived of the tactile-image association we feel is essential to learning applicable ultrasound technique [30]. With a simulator, the student engages in both acquiring the image and interpreting the abnormal finding, while assimilating muscle memory with cognitive learning [31]. This is more reflective of a real clinical setting and enhances the retention of newly learned skills and information [31, 32].

Perhaps the aspect of our course most responsible for its effectiveness was the utilization of an “active learning” format, since this approach has been shown to improve learning outcomes [33]. While several course designs exist, ranging from short introductory sessions to yearlong longitudinal classes, we felt our interactive and multimodal two-day program was time-, resource-, and yield-efficient. To best accomplish our desired educational goals, our course adhered to the following format and order for each organ system: (1) a short 1-2-hour didactic session, (2) demonstration of ultrasound technique and relevant image acquisition by the expert instructor, (3) ultrasound scanning and normal image acquisition by the learner on a healthy volunteer, with direct observation and feedback by the instructor, and (4) pathologic image interpretation utilizing the simulator (Table 1). We believe this format not only is comprehensive, but achieves better skill imprinting by allowing the student to practice it immediately after observing expert didactic sessions and practical simulation.

There are several limitations to our study. The sample was not randomized into two groups (one receiving a course and one not), and hence there was no true control group. However, the number of fellows made this design impractical, and we did not want to deprive our trainees of what we feel is an essential part of critical care education. Second, some of the positive effects of the study at 3 months may be subject to recall bias and varied levels of clinical experience among the fellows. The latter is less likely, however, since our results showed that 2nd- and 3rd-year fellows with more clinical experience did not perform better at baseline than 1st-year fellows. In addition, it is possible that a period longer than 3 months is needed to more appropriately assess the persistence of the positive effects of a course, though we felt that this was a reasonable amount of time for the trainees to have assimilated (or forgotten) their course-related knowledge. The 50 multiple choice questions were the same questions asked throughout the three time points and motivated individuals may score higher after the initial sitting due to further reading material as the study was intended to provide a focal point for learning. Finally, we did not examine the effects of improved ultrasound proficiency on changes in clinical decisions or patient outcomes or the impact of the course on the diverse, ultrasound-naïve critical care faculty that attended as learners. These end points would serve as intriguing targets for future investigations of the benefits of a CCUS course.

## 5. Conclusion

Our results suggest that the introduction of a critical care ultrasound course has both a positive short- and long-term impact on fellows' confidence and proficiency with ultrasound use. Utilizing tools such as written tests to assess basic knowledge, live models to teach practical skills, and ultrasound simulators to teach pathological image identification can help standardize critical care ultrasound training. The proposed course and self-assessment methods presented herein can serve as a model for other institutions looking to implement a formal CCUS curriculum as part of their fellowship training program.

## Figures and Tables

**Table 1:** Two-day critical care ultrasound course curriculum.

Day 1 General critical care ultrasound	

Didactic lectures (2 hours)	
(i) General ultrasound principles (30 minutes)	
(ii) Abdominal vasculature (30 minutes)	
(iii) Hepatobiliary/renal (30 minutes)	
(iv) FAST scan (30 minutes)	
Live demonstration (15 minutes)	
Abdominal vasculature, hepatobiliary, renal, FAST scan	
Hands-on rotation on live model (60 minutes)	
Abdominal vasculature, hepatobiliary, renal, FAST scan	
Pathologic ultrasound simulator cases (60 minutes)	
(i) Case 1: ruptured abdominal aortic aneurysm	
(ii) Case 2: fluid overload: noncollapsible inferior vena cava	
(iii) Case 3: abnormal FAST scan: positive intra-abdominal free fluid	
(iv) Case 4: cholecystitis: thickened gallbladder wall	
(v) Case 5: ascites and renal calculi	
Lunch break (30 minutes)	
Didactic lectures (2 hours)	
(i) Pulmonary ultrasound (60 minutes)	
(ii) Deep vein thrombosis (30 minutes)	
(iii) Vascular access (30 minutes)	
Live demonstration (15 minutes)	
Pulmonary, deep vein thrombosis, vascular access	
Hands-on rotation on live model (60 minutes)	
Pulmonary, deep vein thrombosis, vascular access	
Pathologic ultrasound simulator cases (60 minutes)	
(i) Case 1: right pneumothorax: absence of lung sliding	
(ii) Case 2: right pleural effusion	
(iii) Case 3: pulmonary edema: diffuse B-lines	
(iv) Case 4: right lower extremity DVT: noncompressible vein	

Day 2 Basic critical care echocardiography	

Didactic lectures (1.5 hours)	
(i) Echocardiography: technique and standard views (45 minutes)	
(ii) Common echocardiography applications (45 minutes)	
Live demonstration (30 minutes)	
Demonstration of standard echocardiography views	
Hands-on rotation on live model (60 minutes)	
Practice obtaining standard echocardiography views	
Pathologic ultrasound simulator cases (60 minutes)	
(i) Case 1: pericardial effusion with no tamponade	
(ii) Case 2: depressed left ventricular ejection fraction	
(iii) Case 3: right ventricular strain	
(iv) Case 4: pericardial effusion with tamponade	
Lunch break (30 minutes)	
Didactic lectures (1.5 hours)	
(i) Valvular applications (30 minutes)	
(ii) Hemodynamics (30 minutes)	
(iii) Advanced applications and limitations (30 minutes)	
Live demonstration (30 minutes)	
Hemodynamics and valvular applications	
Hands-on rotation on live model (60 minutes)	
Hemodynamics: cardiac output and ejection fraction assessment	
Hands-on rotation on live model (60 minutes)	
Valvular applications with use of Doppler (color, pulsed, continuous)	

**Table 2:** Baseline test results for the 1st-, 2nd-, and 3rd-year critical care fellows. Results reported as percent ± SD.

	1st year *n* = 4	2nd year *n* = 2	3rd year *n* = 2
Overall written test score	34% ± 6.6%	37% ± 1.0%	40% ± 8.0%
Overall normal image acquisition	20.7% ± 16.1%	40.0% ± 3.7%	38.4 ± 8.0%
Overall pathologic image interpretation	67.6% ± 9.5%	70.2% ± 0.0%	74.4 ± 3.0%

**Table 3:** Comfort level of performing specific ultrasound applications using 5-point Likert scale (1  =  strongly disagree, 2  =  disagree, 3  =  neither disagree nor agree, 4  =  agree, and 5  =  strongly agree). Results reported as median (25th to 75th percentiles). “PRE-CCUS” group was tested within 1 week before CCUS course, “POST-CCUS” group was tested within 1 week after CCUS course, and “3MO-CCUS” group was tested 3 months after CCUS course. ^*∗*^ *p* < 0.05 when compared to PRE-CCUS data.

Median (25%–75%)	PRE-CCUS	POST-CCUS	3MO-CCUS
General bedside ultrasound	2 (1-2)	4 (3-4)^*∗*^	3 (3-4)^*∗*^
Abdominal ultrasound	1 (1-2)	3.5 (3-4)^*∗*^	2.5 (2–3.5)^*∗*^
Pulmonary ultrasound	2 (2–2.5)	4 (4-5)^*∗*^	4 (3.5–4.5)^*∗*^
Lower extremity vascular	1 (1-2)	4 (4-4)^*∗*^	3 (2.5–4)^*∗*^
Echocardiography	2 (2-2)	4 (3-4)^*∗*^	3.5 (3-4)^*∗*^
Central line placement	4 (4-5)	5 (5-5)^*∗*^	5 (5-5)^*∗*^
Peripheral line placement	3.5 (2–4.5)	4.5 (4-5)	4 (3.5–5)
Thoracentesis	4 (1.5–4)	4.5 (3.5–5)	4.5 (3.5–5)

**Table 4:** Number of self-reported ultrasound examinations performed per ICU month at precourse and 3-month follow-up. Results reported as mean ± SD. “PRE-CCUS” group was surveyed within 1 week before CCUS course and “3MO-CCUS” group was surveyed 3 months after CCUS course. ^*∗*^ *p* < 0.05 when compared to PRE-CCUS data.

	PRE-CCUS	3MO-CCUS
General bedside ultrasound	24.5 ± 13.2	58.6 ± 32.8^*∗*^
Abdominal ultrasound	2.0 ± 3.9	7.3 ± 6.8
Pulmonary ultrasound	6.8 ± 7.8	37.8 ± 32.4^*∗*^
Lower extremity vascular	0.13 ± 0.35	6.2 ± 7.1^*∗*^
Echocardiography	7.1 ± 4.4	29.8 ± 23.6^*∗*^
Central line placement	63.6 ± 29.1	67.5 ± 30.1
Peripheral line placement	27.5 ± 45.0	27 ± 40.7
Thoracentesis	6.6 ± 5.3	10.5 ± 7.6
